# Supplementary material for: Statelessness and mental health experiences of Kuwaiti Bidoon people living in the UK: An interpretative phenomenological analysis
Source: PLOS Ment Health. 2026 Feb 11;3(2):e0000441. doi: 10.1371/journal.pmen.0000441 (PMC12893529; doi:10.1371/journal.pmen.0000441)
Supplement: Attachment — Submitted filename: Response letter to reviewers.docx [file pmen.0000441.s002.docx]

10 January 2026

Dear reviewers,

Thank you very much to for the time and effort you have taken to review our manuscript, “*Statelessness and mental health experiences of Kuwaiti Bidoon people living in the UK: An Interpretative Phenomenological Analysis”.* We are very grateful for the helpful comments to improve this manuscript and for the positive comments acknowledging the importance of this piece of research.

Please find below a table outlining the comments we were given, and our response (on the right-hand side), highlighting the changes we have made in response to the suggestions. Please note that **all line numbers below relate to the tracked changes version** of the resubmitted article. A ‘clean’ version has also been provided for ease of reading, but the line numbers may not align.

|  | **Reviewer comment** | **Response** |
| --- | --- | --- |
| **Reviewer 1** | My primary suggestion for revision focuses on broadening the applicability of the article’s results….I think this article unnecessarily limits itself by focusing so narrowly on what these results can show us about the bidoon. The analysis section does link the findings to the broader literature, but I’d like to hear some reflections about what other groups might be similar to the bidoon (other stateless people? other asylum seekers? To what extent do language and culture affect the way mental health issues affect stateless people and asylum seekers?) and how mental health professionals might use this knowledge to work not only with bidoon, but with other people with shared experience. As someone who studies politics and policy, I’d also suggest proposals for changes to the asylum process or other social supports that might help improve the situation for future bidoon asylum seekers, based on this data… | *Thank you so much for your positive feedback and helpful suggestions for improving the article. We have tried to expand the generalisability of the results to stateless groups or displaced people throughout the discussion. Some examples of this are at lines:*   - *611-12* - *618-19* - *633-635* - *666-672*   *There is also a new section entitled “Implications for Policy and Practice” that aims to discuss the implications in a bit more detail, and apply them as broadly as possible to other stateless/displaced people.* |
|  |  |  |
| **Reviewer 2** | Small number of informants for one year of data collection efforts. Would be good to provide more clear information about the recruitment strategy. | *Thank you for raising this; we appreciate it is a small number of participants, but we hope the limitation section of the discussion explains how sensitively recruitment had to be managed, and how much effort was put in to obtain the sample we did manage to recruit. The recruitment methods and strategy were shaped based on four consultation sessions with the UK-based Bidoon community as well as people who work closely with this community. Unfortunately the Kuwaiti Bidoon community, even those residing in the UK, continue to be marginalised and experience persecution and consequences for speaking out against the State of Kuwait. The extent of marginalisation and isolation due to these consequences is outlined in both our introduction and discussion sections. We have also provided some further information in the procedure section to highlight how participants were recruited by partner organisations.* |
|  | The concluding section of the abstract should be strengthened by including clearer and more direct recommendations for both policy and practice, outlining specific actions that could improve healthcare access or support for migrants based on the study’s findings. | *Whilst somewhat constrained by the journal word limit, we have tried to provide a bit more detail about the breadth of issues covered in the discussion/conclusions sections, which we hope now gives readers an initial indication of the key issues covered by the paper.* |
|  | In the introduction or background section, it would be beneficial to add a paragraph describing the rights and entitlements of this social group in the UK, particularly regarding healthcare access, and to provide readers with essential policy context. | *Thank you for highlighting this; we agree with you regarding the importance of providing policy context around the statelessness determination procedures (SDP) and asylum applications and entitlements during the application period. We have now added further detail in lines 136 to 143 regarding the entitlements of stateless people to healthcare and other essential statutory services while awaiting a decision on their SDP application based on Home Office regulations. We have also included detail on the link between lengthy SDP application processes and the risk of destitution due to the lack of entitlements offered to stateless applicants.* |
|  | In the methodology, the reference to partner organisations should be clarified by naming them where possible; if anonymity is required, include a brief justification explaining why this is necessary. The inclusion criteria also need greater detail, specifying whether there was a requirement regarding the length of time participants had lived in the UK and reporting how long they had been in the country. | *An explanation as to why these organisations cannot be named is now included in the procedure section.*  *We have also updated the participant section/inclusion criteria to make it clear that there was no requirement regarding the length of time the person had lived in the UK.*  *We have also added into the procedure section an explanation as to why only minimal demographic information was collected from the participants and therefore why we cannot comment on some of these issues (such as length of time in the UK). This is also highlighted in the limitations section of the discussion.* |
|  | More information about the Arabic interpreter would strengthen transparency—describe how the interpreter was recruited, what training they had received, and the extent of their participation in the wider project. this is briefly mentioned in study limitations. other reflections re- the use of interpreters can be found in the literature | *We agree with you regarding transparency about the interpretation process. We have now amended line 240 to 244 to include information about how the Arabic translator was recruited, their experience working with forcibly displaced and Bidoon community people, as well as the specific training on the aims and objectives of the study that they received from the authors in order to facilitate effective and comprehensive interpretation. Reflections on the use of interpreters and its limitations is outlined in our discussion section.* |
|  | In addition, clarify what the gift voucher given to participants contained and its approximate value. | *Thank you for flagging this; we have now included detail on the reimbursement provided to participants in lines 250 to 252 of the methods section. This includes the remuneration amount, name of the voucher provider, and where it can be redeemed.* |
|  | The results and analysis would benefit from acknowledging that one informant was already a UK citizen, as this likely shaped their mental health experiences differently from participants at other stages of the migration process. Including a brief reflection or analytic layer on this distinction would enrich the interpretation. It would also be valuable to link participant quotes to basic demographic or status information such as age, gender, and migration status, to help readers contextualize perspectives and strengthen analytical depth. | *You have raised a salient point; thank you for highlighting this as it points to an important area that did not emerge organically in this study. We agree with you that having a ‘stable’ immigration status such as being granted leave to remain (UK specific) or citizenship might be experientially different to someone currently living with an uncertain immigration status. We considered this at the methodology, design, and initial analysis stages but eventually focused largely on the transition and settling-in process as these are the topics that emerged organically from the interviews. From literature that we draw on in the introduction section regarding the SDP application process, we have also identified that the route to citizenship after being granted leave to remain is also not straightforward. The difference in permanence of immigration statuses is a promising avenue for future research with newly arrived and long-term settled stateless people.*  *We have added a point about this into the limitations section (lines 791-800) and there is also some mention of the relevance of this issue at line 644 in the discussion. We hope that this will suffice to fulfil your recommendations at this stage.*  *With regards to contextualising information, we have already listed the respondents immigration statuses in the ‘participants’ segment of the methods section but specific demographic details such as the participants age, gender, etc. has been intentionally omitted to protect the anonymity of respondents. We want to emphasise this decision was made in light of the historical and active threats and consequences the Bidoon community face if they speak out about their experience of statelessness. As this is a very small community residing in the UK, we felt it was necessary to protect their anonymity to minimise any risk of repercussions (even if these might only be perceived, their level of fear is understandably high) from being involved in our study.* |
|  | In the discussion, consider incorporating recent literature on the suitability and capability of UK mental health services to meet the needs of vulnerable migrants. Relevant studies include work published in the [Journal of Ethnic and Migration Studies (2023](https://www.tandfonline.com/doi/full/10.1080/1369183X.2023.2181126)) and [BMJ Open (2025),](https://bmjopen.bmj.com/content/15/6/e096267) or both of which explore implications for interventions and service delivery. Integrating insights from these studies will help situate your findings within existing research. | *Thank you also for sharing these relevant articles and raising this important point about the applicability and generalisability of our findings in the context of other forcibly displaced populations in the UK. The articles have been very insightful for us in contextualising our findings and we have made reference to these at various points in the discussion section (e.g. 700-708). We have also tried to make clearer the discussion about limitations of existing service provision and discuss recommendations in more detail – we have included a new section entitled “Implications for Practice and Policy) to highlight this part of the paper more clearly.* |
|  | Furthermore, it would be helpful to indicate where participants lived and reflect on how regional socio-economic and political environments across the UK—such as life in sanctuary cities compared with less welcoming areas—shape migrant experiences. | *As above, we collected only the bare minimum demographic data from participants, in order to protect their identity and ameliorate their concerns about anonymity. This is now clearly stated in the procedure section of the methods part of the paper. As a result, we are unable to comment on this issue relating to the client’s regional location in the UK, which was unknown to us as all participants opted for an online interview. This is an interesting point though, which we have now included for reflection in the limitations section of the discussion.* |
|  | Finally, the paper should elaborate on the implications of these findings for service provision. Drawing on both NHS and NGO contexts, highlight how mental health support structures could be improved, what barriers persist, and how the study’s insights can inform better coordination and access for migrants. This addition will ensure the manuscript makes a strong and practical contribution to policy and service improvement debates. | *Thank you for highlighting this – as above, we have inserted a new heading “Implications for Practice and Policy” to our discussion section to draw attention to the recommendations that flow from the findings of this article. We have tried to strengthen and elaborated on the recommendations as you have suggested*. |
|  |  |  |
| **Other changes required by journal editor** | Please include a complete copy of PLOS’ questionnaire on inclusivity in global research in your revised manuscript. Our policy for research in this area aims to improve transparency in the reporting of research performed outside of researchers’ own country or community. The policy applies to researchers who have travelled to a different country to conduct research, research with Indigenous populations or their lands, and research on cultural artefacts. The questionnaire can also be requested at the journal’s discretion for any other submissions, even if these conditions are not met.  Please find more information on the policy and a link to download a blank copy of the questionnaire here:  <https://journals.plos.org/mentalhealth/s/best-practices-in-research-reporting>. Please upload a completed version of your questionnaire as Supporting Information when you resubmit your manuscript. | *This questionnaire has been completed and uploaded as requested.* |
|  | Please ensure that your Ethics Statement is available in its entirety at the beginning of your Methods section, under a subheading 'Ethics Statement'. It must include:  1) The name(s) of the Institutional Review Board(s) or Ethics Committee(s)  2) The approval number(s), or a statement that approval was granted by the named board(s)  3) (for human participants/donors) - A statement that formal consent was obtained (must state whether verbal/written) OR the reason consent was not obtained (e.g. anonymity). | *This can be found in the methods section of the paper. We have now put the ethics statement under its own separate heading.* |
|  | 3.We note that you have indicated that there are restrictions to data sharing for this study. For studies involving human research participant data or other sensitive data, we encourage authors to share de-identified or anonymized data. However, when data cannot be publicly shared for ethical reasons, we allow authors to make their data sets available upon request. For information on unacceptable data access restrictions, please see <http://journals.plos.org/plosone/s/data-availability#loc-unacceptable-data-access-restrictions>.   a) If there are ethical or legal restrictions on sharing a de-identified data set, please explain them in detail (e.g., data contain potentially identifying or sensitive patient information, data are owned by a third-party organization, etc.) and who has imposed them (e.g., a Research Ethics Committee or Institutional Review Board, etc.). Please also provide contact information for a data access committee, ethics committee, or other institutional body to which data requests may be sent.   b) If there are no restrictions, please upload the minimal anonymized data set necessary to replicate your study findings to a stable, public repository and provide us with the relevant URLs, DOIs, or accession numbers. Please see <http://www.bmj.com/content/340/bmj.c181.long> for guidelines on how to de-identify and prepare clinical data for publication. For a list of recommended repositories, please see <https://journals.plos.org/plosone/s/recommended-repositories>. You also have the option of uploading the data as Supporting Information files, but we would recommend depositing data directly to a data repository if possible.  Please update your Data Availability statement in the submission form accordingly. | *We have detailed in the methods section of the paper (Data and Analysis section) why we cannot share this data in an unrestricted way i.e. due to the highly sensitive nature of it, and the vulnerability of the participants. I note that this position has been endorsed by one of the reviewers, who shared that they also have experience of completing research alongside this community. We are willing to share the data to fulfil requests that have an important purpose, and upon being able to verify the credentials of any academic professional making a request. The data can be obtained via the corresponding author. A data availability statement has also been added to the manuscript to this effect (lines 837-841).* |

We do hope that this article now meets the expected standard for publication, but we welcome any further feedback that you or the reviewers might have for us at this stage.
